# Prognostic role of Glasgow prognostic score in patients with colorectal cancer: evidence from population studies

**DOI:** 10.1038/s41598-017-06577-2

**Published:** 2017-07-21

**Authors:** Yangyang Liu, Xingkang He, Jie Pan, Shujie Chen, Liangjing Wang

**Affiliations:** 1 Department of Gastroenterology, Second Affiliated Hospital, Zhejiang University School of Medicine, Hangzhou, Zhejiang 310009 China; 2 Institute of Gastroenterology, Zhejiang University, Hangzhou, Zhejiang 310009 China; 3 Department of Gastroenterology, Sir Runrun Shaw Hospital, Zhejiang University School of Medicine, Hangzhou, Zhejiang 310016 China; 4 Department of Endocrinology and Metabolism, Second Affiliated Hospital, Zhejiang University School of Medicine, Hangzhou, Zhejiang 310009 China

## Abstract

Glasgow prognostic score (GPS) has been reported to be an indicator of prognosis for various cancers. However, the relationship between GPS and colorectal cancers (CRC) remains unclear. A comprehensive search of Pubmed, Embase, Cochrane library, Web of Science, ChinaInfo and Chinese National Knowledge Infrastructure was performed to identify eligible studies, from which the risk of overall survival (OS) and cancer-specific survival (CSS) were extracted. A random-effect model was adopted to combine hazard ratio (HR) and 95% confidence interval (CI). 25 articles with a total of 5660 participants were included. The pooled results indicated that elevated GPS was associated with poor OS (HR = 2.83, 95%CI: 2.00–4.00, *P* < 0.01) and CSS (HR = 1.94, 95%CI: 1.51–2.49, *P* < 0.01). This correlation was confirmed both in primary operable and advanced inoperable patients. Increased GPS was also closely related to advanced tumour-node-metastasis (TNM) stage (odds ratio [OR] = 1.44, 95% CI: 1.010–2.065, P < 0.05) and elevated level of serum carcinoembryonic antigen (OR = 2.252, 95% CI: 1.508–3.362, *P* < 0.01). Subgroup analysis revealed a significant association between high GPS and poor survival outcome according to the factors of sample size, study of region and cut-off value of GPS level. These findings suggest that GPS may serve as a reliable predictive index for patients with CRC.

## Introduction

Colorectal cancer (CRC) is the third most common cancer worldwide and accounts for 10% of all newly diagnosed cancers^1^. Although the surgical techniques, chemotherapy and molecular-target therapy have dramatically developed, the long-term survival rate of patients with CRC remains low, particularly in patients with advanced stage cancers^2^. Accurate prediction of prognosis will assist in adopting appropriate therapies and contribute to better management of CRC patients. Currently, the tumour-node-matastasis (TNM) surgical staging system, based on postoperative histopathology of the tumour, is considered to be the golden standard for predicting clinical outcomes of cancer patients^3^. However, its accuracy has been debated as tumour progression may not be solely determined by the characteristics of the tumour, but the host inflammatory responses as well^4–6^. Therefore, in recent years, great efforts have been made to identify inflammation-related factors for precise prediction of disease prognosis.

Glasgow Prognostic Score (GPS) is such an inflammation-based factor, defined by the combination of the level of serum C-reactive protein (CRP) and albumin, which are indicators of systematic inflammatory response and nutritional status respectively^7^. The first study on GPS reported by Forrest *et al*.^8^ in 2003 showed that it could be an predictor of prognosis for non-small-cell lung cancer patients. Subsequently, growing evidence suggested that GPS was served as an independent prognostic index in a variety of malignant cancers including hepatocellular carcinoma^9^, esophageal cancer^10^, gastric cancer^11^, renal cancer^12^, and pancreatic cancer^13^. For patients with CRC, the GPS system was also widely studied, but the results were controversial. Leitch and his colleagues^14^ reported that GPS was an independent prognostic factor for CRC, while Son *et al*.^15^ did not detect significant association between GPS and patients’ survival. This makes it rational to perform systematic evaluation of the correlation between GPS and prognosis of patients with CRC to further clarify its clinical significance.

## Results

### Characteristics of the enrolled studies

**Figure 1:**
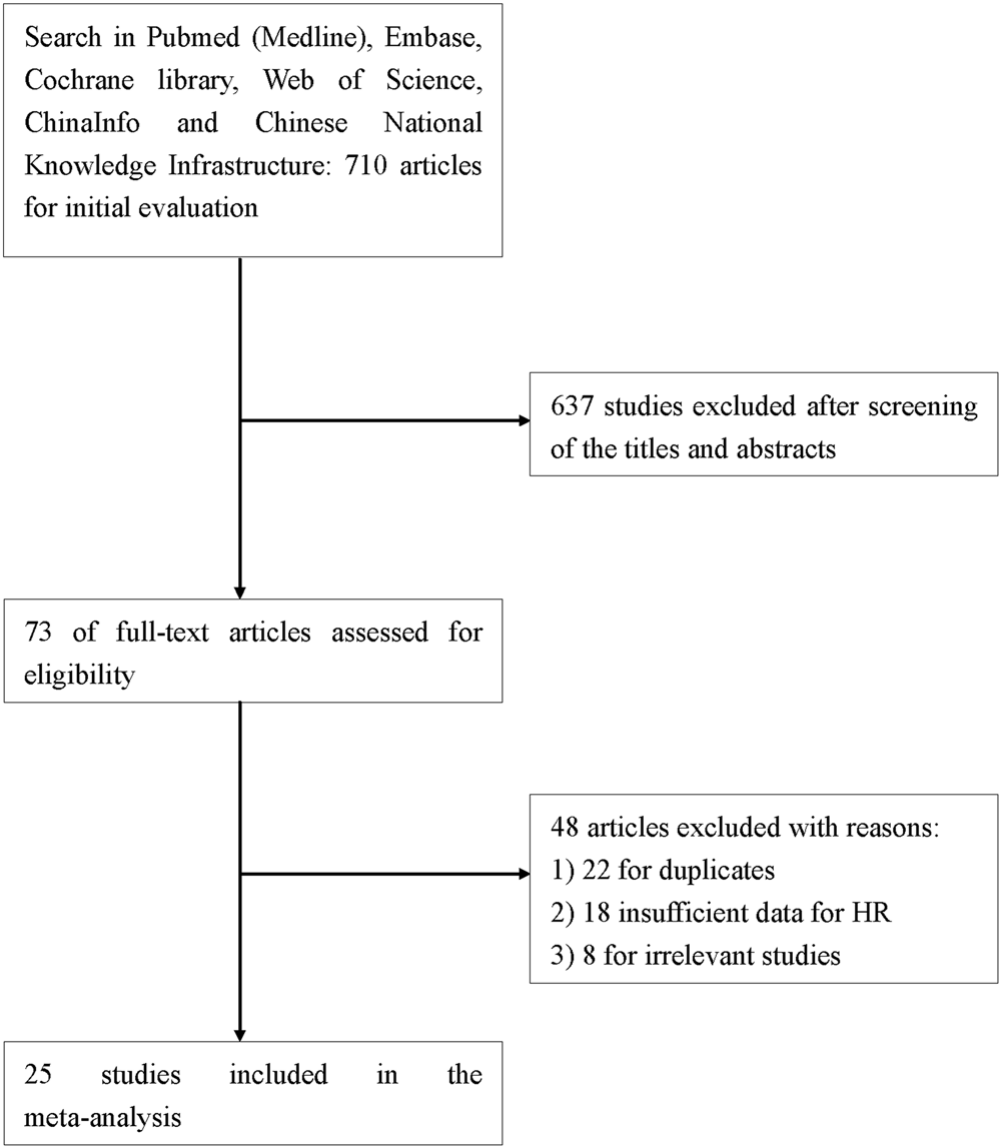
Flow diagram of the study selection process.

As shown in Fig. 1, a total of 710 records were identified from different databases by initial search. After screening the titles and abstracts, 73 full-text articles were assessed for further eligibility analysis. 48 of them were subsequently excluded as follows: 18 studies without sufficient data; 22 of overlapping populations; 8 of only documented with abstracts, comments or reviews. Finally, 25 original reports published between the year 2006 and 2016 with sample size ranging from 42 to 1000 were enrolled into the meta-analysis^14–38^. The characteristics of all the studies were summarized in Table 1.

**Table 1:** Characteristics of included studies.

First Author	Year	Study region	Number (M/F)	Age (years)	Treatment	Follow-up	Stage
Read^16^	2006	Australia	51 (30/21)	64 (40–79)^a^	chemotherapy	Median: 29.8 months	IV
Ishizuka^17^	2007	Japan	315 (186/129)	<70: 162; ≥70: 153	surgery	11 days-13.7 months	0-IV
Xiao^22^	2013	China	223 (148/75)	<65: 181; ≥65: 42	Surgery	43 (6–84) months	I-IV
Kobayashi^24^	2014	Japan	106 (77/29)	<70: 66; ≥70: 40	Surgery	Median: 38 m	IV
Nozoe^25^	2014	Japan	272 (160/112)	70.4 (24–90)^a^	Surgery	NR	I–IV
Lin^26^	2015	China	99 (35/64)	62.63 ± 10.86^b^	Surgery	Median: 60 months	II
Shibutani^27^	2015	Japan	254 (139/115)	60 (26–86)^a^	surgery	NR	II、III
Ishizuka^28^	2016	Japan	627 (400/227)	67.75 ± 11.69^b^	Surgery	NR	0–IV
Eren^29^	2016	Turkey	115 (64/51)	66.1 ± 12.77^b^	Surgery	20 (7–41) months	I–IV
Kishiki^32^	2013	Japan	79 (42/37)	≤70: 43; >70: 36	Surgery	32 (1–66) months	IV
Son^15^	2013	Korea	624 (368/256)	<60: 295; ≥60: 329	Surgery	42 (1–66) months	I–III
Adachi^35^	2015	Japan	65 (37/28)	64 (17–83)^a^	surgery	NR	I–IV
Ghanim^36^	2015	Austria	52 (31/21)	62.7 ± 11.4^b^	surgery	NR	IV
Song^37^	2015	Koea	177 (83/94)	52 (25–81)^a^	Korean treatmen	3.1 (0.1–33.3) months	IV
Toiyama^30^	2011	Japan	219 (136/83)	66 (58–73)^a^	surgery	52.7 (56.9 63.8) months	II, III
Park^38^	2016	UK	1000 (548/452)	<65: 330; 65–74: 347; >75: 323	surgery	56 (10–206) months	0–III
Choi^23^	2014	Korea	105 (63/42)	63 (32–86)^a^	Surgery	44 (2–81) months	I–IV
Leitch^14^	2007	UK	233 (129/104)	<65: 34; 65–74: 27; >75: 23	Surgery + chemotherapy	12 (6–73) months	I–IV
Manabu^31^	2012	Japan	42 (26/16)	<70: 12; ≥70: 30	chemotherapy	Median: 424 days	IV
Inoue^33^	2013	Japan	245 (146/99)	64 (29–85)^a^	chemotherapy	NR	IV
Nakagawa^34^	2014	Japan	343 (219/124)	62.83 ± 3.85	surgery	NR	IV
Kobayashi^18^	2010	Japan	63 (44/19)	<70: 41; ≥70: 22	surgery	38 (30.5–45.6) months	I–IV
Furukawa^19^	2012	Japan	40 (30/10)	66.1 ± 9.7^b^	chemotherapy	NR	IV
Sugimoto^20^	2012	Japan	366 (209/157)	≤70: 240; >70: 126	surgery	Median: 70.8 months	II, III
Madea^21^	2013	Japan	94 (51/43)	<70: 62; ≥70: 32	surgery	Median: 21 months	IV
**First Author**	**Survival analysis**	**Cut-off value**	**Lymphatic invasion (+/−)**	**Nevous invasion (+/−)**	**CEA (ng/ml)**	**Differentiation (well/moderate/poor)**	
Read^16^	OS	1	NR	NR	NR	NR	
Ishizuka^17^	OS	1	NR	NR	<6: 185; ≥6: 120	NR	
Xiao^22^	OS	1	NR	NR	<5: 127; ≥5: 96	NR	
Kobayashi^24^	OS	1	75/31	96/10	<30: 75; ≥30: 56	Well: 68; moderate & poor: 38	
Nozoe^25^	OS	2	112/160	65/207	NR	82/170/20	
Lin^26^	OS	2	NR	27/72	≤10: 73; >10: 26	25/50/24	
Shibutani^27^	OS	1	184/47	68/170	≤5: 154; >5: 44	Well & moderate: 234; poor & mucinous: 19	
Ishizuka^28^	OS	2	129/498	131/496	≤8.7: 433; >8.7: 194	Well or moderate: 583; others: 44	
Eren^29^	OS	2	68/47	NR	<5: 97; ≥5: 18	13/81/11	
Kishiki^32^	OS	2	NR	NR	<6: 19; ≥6: 60	Well or moderate: 77; others: 2	
Son^15^	OS	2	NR	NR	<5: 450; ≥5: 172	Low: 562; high: 62	
Adachi^35^	OS	2	NR	NR	<10: 25; ≥10: 40	Well & moderate: 45; poor & undifferentiated: 14; unknown: 6	
Ghanim^36^	OS	1	NR	NR	NR	NR	
Song^37^	OS	1	NR	NR	≤5: 31; >5: 140	NR	
Toiyama^30^	OS、CSS	1	191/28	96/123	≤6: 134; >6: 85	Differentiated: 200; non-differentiated: 19	
Park^38^	OS、CSS	1	NR	507/493	NR	Well & moderate: 894; poor: 96	
Choi^23^	CSS	2	NR	NR	<5: 63; ≥5: 41	Well & moderate: 59; poor: 42	
Leitch^14^	CSS	1	NR	NR	NR	NR	
Manabu^31^	CSS	2	NR	NR	NR	NR	
Inoue^33^	CSS	2	NR	NR	NR	Differentiated: 219; non-differentiated: 26	
Nakagawa^34^	CSS	1	NR	NR	124.4 ± 116.6	Moderate: 203; others: 128	
Kobayashi^18^	CSS	1	49/14	53/10	<30: 43; ≥30: 18	Well: 51; moderate & poor: 12	
Furukawa^19^	CSS	2	NR	NR	<100: 22; ≥100: 18	NR	
Sugimoto^20^	CSS	2	362/4	343/23	≤3: 154; >3: 212	Well: 162; others: 204	
Madea^21^	CSS	2	NR	NR	NR	Wel & moderate: 79; others: 15	

NR = not reported, OS = overall survival, CSS = cancer-specific survival.

^a^Mean (range).

^b^Mean ± SD.

Among the included studies, some patients were enrolled multiple times for different purposes in publications. In Leitch and his colleagues’ research^14^, the patients with TNM stage I,IIand III were overlapped with those in Park’s study^38^. So we only extracted the data of patients with stage IV from the prior study. Among all the studies, 15 were conducted in Japan, 2 in the United Kingdom, 2 in China, 3 in Korea, 1 in Australia, 1 in Turkey and 1 in Austria. Ten studies only included patients with stage IV, one only included stage II and the remaining included a mixed population. The cut-off values of GPS were diverse. In 12 studies, GPS of 1 was defined as elevation, while in the remaining 13 literatures, GPS of 2 was considered as an increase. Surgery was the main treatment approach in 20 of the 25 included studies. A ‘Korean treatment’ was discussed in one study conducted in Korea, and the remaining four studies focused on chemotherapy.

### GPS and survival outcome

**Figure 2:**
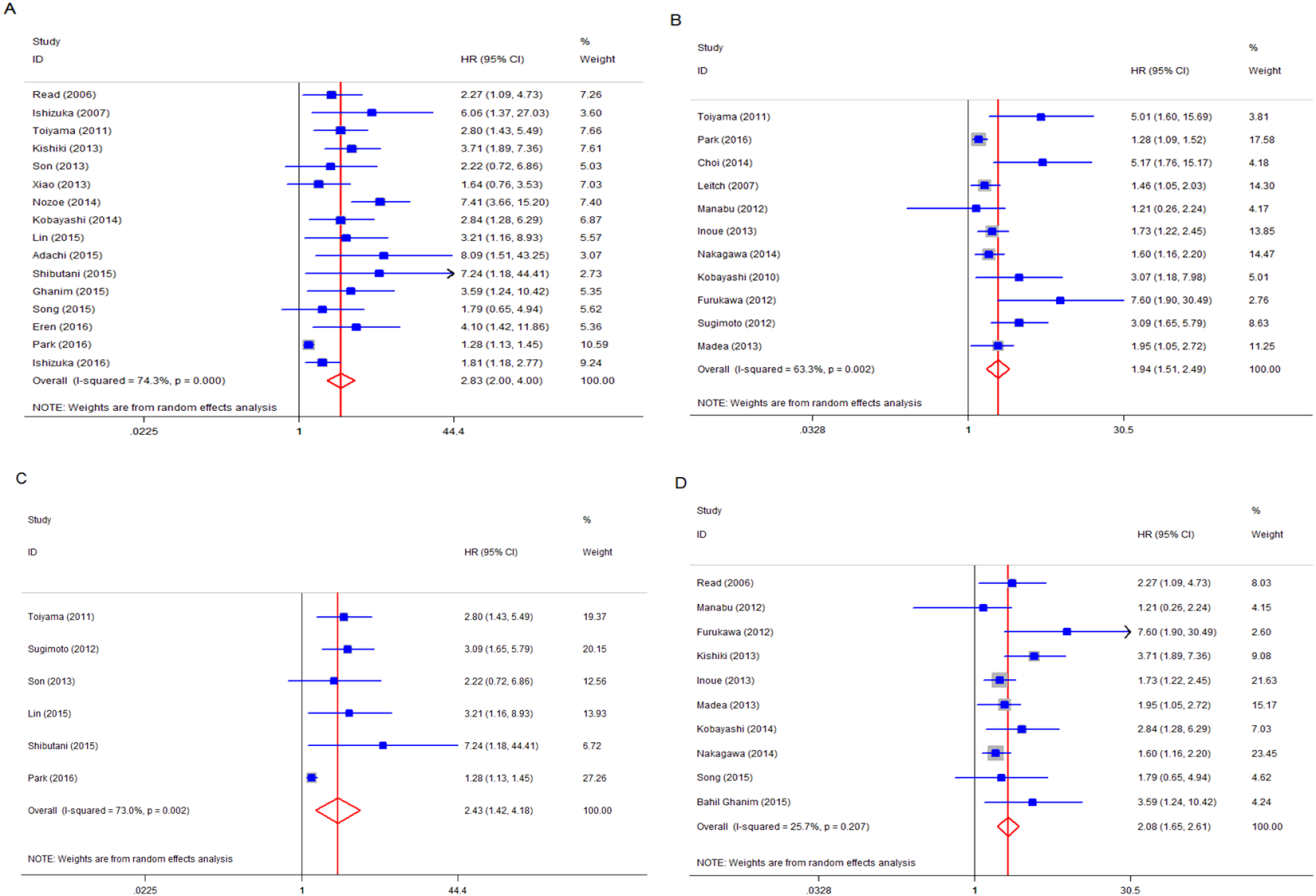
(**A**) Forest plot of hazard ratio for the association between GPS and OS in patients with CRC; (**B**) Forest plot of hazard ratio for the association betweenGPS and CSS; (**C**) Forest plot of hazard ratio for the association between elevated GPS and survival in patients with primary operable disease; (**D**) Forest plot of hazard ratio for the association between elevated GPS and survival in patients with advanced inoperable diseases.

Obvious heterogeneity was detected in this meta-analysis (OS: *I* ^*2*^ = 74.3%, *P* < 0.001; CSS: *I* ^*2*^ = 63.3%, *P* = 0.002), so the random-effect model was adopted to calculate the pooled HR and 95% CI values. There were 16 studies reporting the relationship between pretreatment GPS and OS in CRC patients. The pooled HR of 2.83 (95% CI: 2.0–4.0, *P* < 0.001) implied that CRC patients with elevated GPS were expected to have poor OS (Fig. 2A). There were 11 studies presenting the relationship between GPS and CSS. The pooled result showed significant association between elevated GPS and short CSS (HR = 1.94, 95% CI: 1.51–2.49, *P* < 0.001) (Fig. 2B). Furthermore, subgroup analysis was conducted, stratified by sample size, study of region and cut-off value of GPS. As shown in Table 2, increased GPS was also predicted to have a significant poor prognostic effect on survival in CRC patients in those subgroups.

**Table 2:** Results of subgroup analysis.

Subgroup	No. of studies	HR (95% CI)	P value	Heterogeneity	
				*I* ^*2*^	Ph
Overall survival					
Sample size					
>500	3	1.450 (1.100–1.913)	0.008	36.3%	0.208
≤500	13	3.284 (2.510–4.297)	<0.001	12.1%	0.323
Study of region					
Japan	8	3.628 (2.344–5.615)	<0.001	53.7%	0.034
others	8	2.038 (1.402–2.963)	<0.001	50.3%	0.050
Cut-off value of GPS					
1	9	2.286 (1.533–3.409)	<0.001	61.9%	0.007
2	7	3.506 (2.158–5.695)	<0.001	57.0%	0.030
Cancer-specific survival					
Sample size					
>500	1	1.280 (1.084–1.512)			
≤500	10	2.098 (1.612–2.729)	<0.001	48.9%	0.0728
Study of region					
Asia	9	2.29 (1.697–3.090)	<0.001	46.8%	0.082
others	2	1.315 (1.133–1.525)	<0.001	0%	<0.001
Cut-off value of GPS					
1	5	1.589 (1.216–2.076)	0.001	56.5%	0.057
2	6	2.365 (1.599–3.500)	<0.001	48.5%	0.084

In addition, we explored the influence of GPS on survival in different disease stages. By grouping CRC patients into two subsets, 6 studies included patients with primary operable diseases (stages I,II, III), and 10 studies included patients with advanced inoperable diseases (stage IV). The rest included a mixed population and were excluded as the data was not able to be extracted according to the above two groups. The pooled estimate also showed that elevated GPS was correlated with worse survival, both in primary operable patients (HR = 2.43, 95% CI: 1.42–4.18, *P* = 0.001) (Fig. 2C) and advanced inoperable patients (HR = 2.08, 95% CI: 1.65–2.61, *P* < 0.001) (Fig. 2D).

### GPS and clinicopathological features

**Figure 3:**
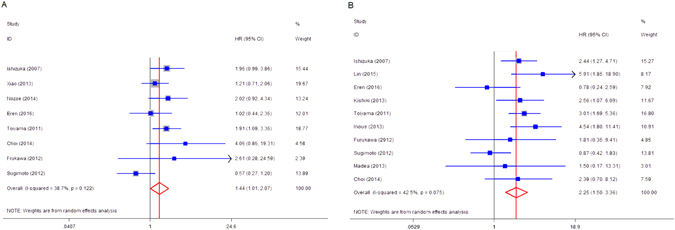
The forest plot between pretreatment GPS and clinicopathological parameters in CRC. (**A**) TNM stage (III, IV vs 0, I, II); (**B**) serum CEA.

There were 8 studies reporting the relationship between the level of GPS and TNM stage^16–18, 20, 21, 23, 27, 29^. The combined odds ratio (OR) of 1.44 (95% CI: 1.010–2.065, *P* = 0.044) displayed that patients with elevated GPS was prone to be in advanced TNM stages, with no significant heterogeneity (*I* ^*2*^ = 38.5%, *P* = 0.123; Fig. 3A). The relationship between GPS and serum carcino-embryonic antigen (CEA) level in CRC patients was presented in 10 studies^16–19, 21, 24, 27, 29, 31, 33^. The pooled OR was 2.252 (95% CI: 1.508–3.362, *P* = 0.000), indicating that elevated GPS was associated with elevated serum CEA, with no obvious heterogeneity (*I* ^*2*^ = 41.9%, *P* = 0.078; Fig. 3B). There was no significant correlation between increased GPS and lymphatic invasion (positive vs negative) and vascular invasion (positive vs negative) (Table 3).

**Table 3:** Relationship between pretreatment GPS and lymphatic invasion and venous invasion.

Factors	OR	95% CI	*P*-value
Lymphatic invasion	1.634	0.788–3.388	0.187
Venous invasion	1.295	0.846–1.981	0.233

### Meta-regression analysis

**Figure 4:**
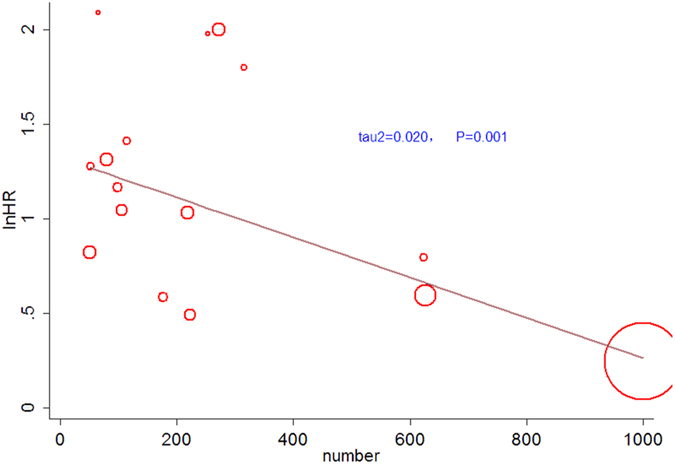
Meta-regression plot of HRs of OS against sample size.

In order to explore the source of heterogeneity, a meta-regression analysis was performed based on the following variables: sample size, study region, cut-off value of GPS and TNM stage. The result showed that for the 16 studies on OS, sample size was significantly related to heterogeneity (*P* = 0.001) (Fig. 4). Study region, cut-off value and TNM stage were negatively related to hazards of endpoints (Table 4). For 11 studies on CSS, study of region was significantly related to heterogeneity, while sample size, cut-off value and TNM stage were negatively related to hazards of endpoints (Table 4).

**Table 4:** Results of meta-regression on OS and CSS

Variables	Coeffecient	Standard error	t	*P* value	95% CI
Overall survival					
Cut-off value	0.4296658	0.2943846	1.46	0.166	−0.2017264, 1.061058
Sample size	−0.001059	0.0002384	−4.44	0.001	−0.0015702, −0.0005477
region	0.2468601	0.3658075	0.67	0.515	−0.5682097, 1.06193
TNM stage	0.7961549	0.7037753	1.13	0.291	−0.8267539, 2.419064
Cancer-specific survival					
Cut-off value	0.3533804	0.2600391	1.36	0.207	−0.234869, 0.9416297
Sample size	−0.0005666	0.0004215	−1.34	0.212	−0.00152, 0.0003868
region	0.3859487	0.1528578	2.52	0.036	0.033458,0.7384393
TNM stage	0.6038852	0.4511961	1.34	0.223	−0.4630241,1.670795

### Sensitivity analysis

**Figure 5:**
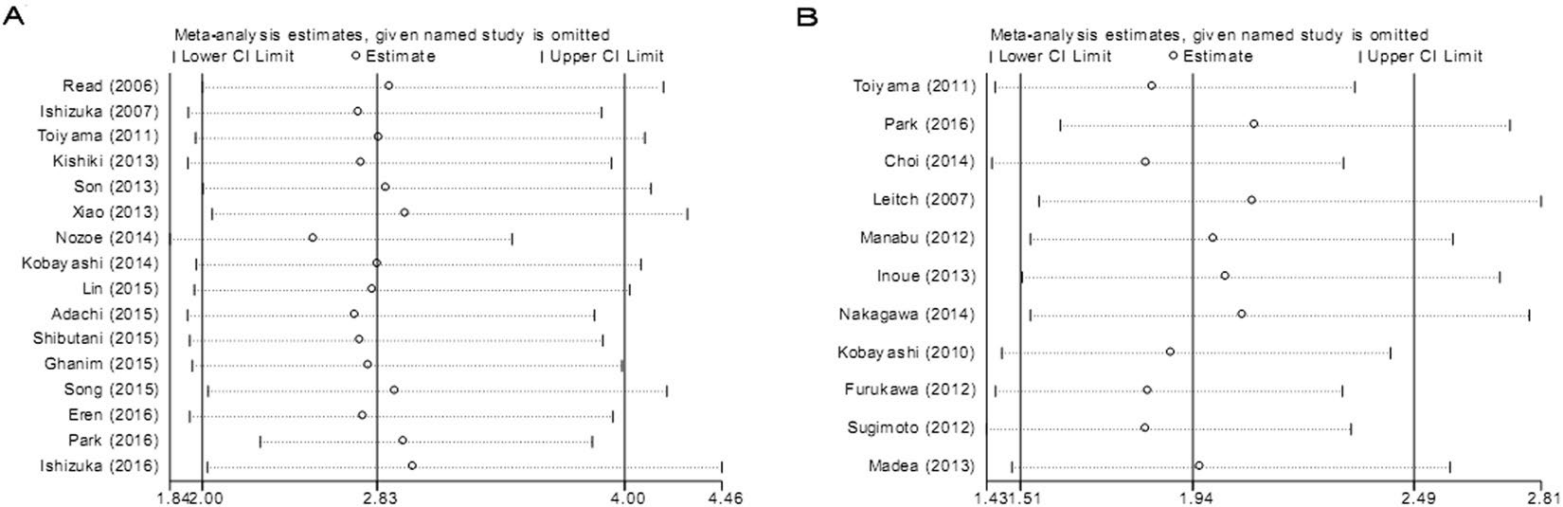
(**A**) Sensitivity analysis of 16 studies inclued in this meta-analysis for OS. (**B**) Sensitivity analysis of 11 studies inclued in this meta-analysis for CSS.

In order to assess whether the results were credible and stable with obvious heterogeneity, sensitivity analysis was carried out by means of omitting each study by turns (Fig. 5A and B). The result indicated that there was no obvious influence of one individual study on the pooled HR.

### Publication bias

**Figure 6:**
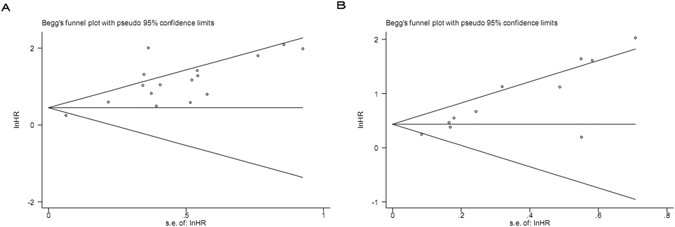
(**A**) Funnel plot of 16 included studies in this meta-analysis for OS; (**B**) Funnel plot of 11 included studies in this meta-analysis for CSS.

A funnel plot and Egger’s test were employed to investigate publication bias. As a result, for the studies of the GPS and OS, Begg’s test showed no publication bias (*P* = 0.260, Fig. 6A), whereas, Egger’s test revealed statistical significance (*P* < 0.001). For the studies of the GPS and CSS, substantial publication bias was detected both in the Begg’s test (*P* = 0.013, Fig. 6B) and Egger’s test (*P* < 0.001). Consequently, the “trim and fill” analysis was further performed and the recalculated result did not change significantly (OS: HR = 1.381, 95% CI: 1.247–1.529, *P* < 0.001; CSS: HR = 1.430, 95% CI: 1.279–1.599, *P* < 0.001), indicating the stability of our pooled results.

## Discussion

Inflammation plays an important role in the development and progression of various tumours. Cancer can induce local or systematic inflammation, mediated by the activation of transcription factors and release of cytokines, which can inversely influence tumour activities, including cell proliferation, angiogenesis, cell migration, and invasion^39, 40^. There are several inflammation-based prognostic systems having been reported in cancers. GPS is one of them, combining serum CRP and albumin which are closely connected with the prognosis of cancer patients respectively^41, 42^. Initially, GPS was applied to determine the prognosis of patients with inoperative lung cancer^8^. Thereafter, it was increasingly used to assess the outcome of patients with multiple malignant tumours^43–47^. Recently, a number of investigations attempted to evaluate the significance of GPS in predicting patients’ survival in CRC^14–38^, but had the inconsistent results.

The current study, to our best knowledge, is the most comprehensive meta-analysis assessing the correlation between GPS and the prognosis of patients with CRC. A total of 25 studies containing 5660 CRC patients were included. As shown in this meta-analysis, the pooled HR of OS and CSS were 2.83 (95% CI: 2.00–4.00, *P* < 0.001) and 1.94 (95% CI: 1.51–2.49, *P* < 0.001) respectively, which suggested that patients with elevated GPS were predisposed to exhibit poor survival outcome. When subgroup analysis was performed stratifying by sample size, study of region and cut-off value of GPS, worse survival was also presented in CRC patients with elevated GPS in all the subgroups, which suggested that our results were reliable.

As staging is currently the most important prognostic indicator for CRC, we grouped patients into two subsets–the primary operable diseases and the advanced inoperable diseases. Then we conducted meta-analysis in these two groups respectively, regarding the information with reference to GPS and survival outcome. Inspiringly, the pooled results were consistent in the two groups, despite their different presentations, diagnosis and treatments, both suggesting that elevated GPS was associated with worse survival in CRC patients, which further validated our conclusion.

Furthermore, an obvious relationship between GPS and clinical parameters, including TNM stage and serum CEA level was observed, which further confirmed that GPS could be a promising predicting index for CRC patients. However, we found no significant correlation between the increased GPS and lymphatic and venous invasion. One possible explanation could be that among the included studies, only 8 studies had reported the connection between GPS and lymphatic invasion, 9 between GPS and venous invasion, resulting in a small sample size for analyzing.

The forest plot revealed heterogeneity in this meta-analysis (*I* ^*2*^ = 74.3%, *P* < 0.001; *I* ^*2*^ = 63.3%, *P* = 0.002). Therefore, we performed meta-regression analysis to explore the source. The results indicated that the sample size might contribute to the heterogeneity across 16 studies on OS, while the region of study contributed to heterogeneity across 11 studies on CSS. Among the included 25 studies, only 3 contained with populations more than five hundreds. A small sample size was usually considered as the source of heterogeneity. Of note, the majority of the included studies (19/25) were conducted in Asian medical institutions, and ethnic background and life styles may contribute to the variations in cancer patients’ prognosis, which was consistent with other meta-analysis^48^. However, the subgroup analysis in terms of the sample size and study region did not alter the overall results.

There are several limitations that should be considered in this meta-analysis. First of all, though the amount of included studies was large, significant interstudy heterogeneity was still observed and could not be eliminated completely. The variables included in our meta-regression analysis partly explained the heterogeneity, other factors might affect the prognosis. Secondly, most of the included studies were retrospective. Thus, further larger scale, well-designed prospective investigations are required in the future.

In conclusion, current evidence from the meta-analysis identifies elevated GPS as a promising prognostic biomarker in CRC. GPS, calculated from two conventional laboratory data, does not demand additional examinations such as imaging techniques or histopathology, and could be easily obtained from peripheral blood test. Additionally, compared to the existing evaluation system such as TNM stage, GPS could be obtained before operation. Pre-operative elevated GPS suggests patients at high risk of recurrence and poor survival, which could help doctors to adopt more careful surgery and more rigorous follow-up.

## Materials and Methods

### Literature search

We performed electronic literature searches using Pubmed, Embase, Cochrane library, Web of Science, ChinaInfo and Chinese National Knowledge Infrastructure before June 01, 2016. Following key search terms were included in our study: “Glasgow Prognostic Score”, “GPS”, “prognosis”, “colorectal carcinoma”, “colon cancer”. The references of all included studies were screened to identify additional related full articles.

### Study selection

A study was considered eligible according to the following criteria: (1) the study was written in English or Chinese; (2) the diagnosis of CRC was confirmed by pathology; (3) correlation between GPS and overall survival (OS)/cancer-specific (CSS) was presented in the article; (4) HRs with its 95% CI were reported in the study. Exclusion criteria was defined as following: non-human CRC studies; studies published in abstracts, letters, reviews, case reports, expert comments, duplicated articles, and studies without HR and 95% CI.

### Data extraction

Two investigators (LYY and HXK) independently extracted data from the eligible studies and disagreements were resolved by discussion. The following items were obtained from each study: (1) name of the first author, study region, publication year, number of cases, age, dominating treatment, follow-up period; (2) clinicopathological parameters including TNM stage, lymphatic invasion, venous invasion, lymph node metastasis, and carcinoembryonic antigen (CEA) level; (3) survival data of OS and CSS; (4) the cut-off value of GPS.

### Quality assessment

We evaluated the articles identified by the above criteria and implemented quality assessment according to the Newcastle-Ottawa-Scale (NOS)^49^. Any discrepancy was resolved by consensus.

### Statistical analysis

Data analysis was conducted using Stata 12.0 (Stata Corporation, Texas, US). The HRs with its 95% CI was directly obtained from each literature and the pooled HR was used to assess the significance of GPS on OS and CSS of the patients with CRC. If several estimates were reported for the same value, HRs and 95% CI were extracted preferentially from multivariate analysis where available. Otherwise, HRs were extracted from univariate analysis. For analyzing the correlation between GPS and clinicopathological parameters, OR with its 95% CI was combined by Mantel-Haenszel method as the effective value. Heterogeneity among pooled results was assessed using Cochrane *Q* test and Higgin *I* ^*2*^ statistic. *P* < 0.05 or/and *I* ^*2*^ > 50% were considered statistically heterogeneous and the random-effect model was applied; otherwise, the fixed-effect model was performed. Publication bias was evaluated using the funnel plot and Egger’s test (values of *P* > 0.05 indicated lack of publication bias)^50^. Meta-regression analysis was conducted to explore potential sources of heterogeneity. We also performed a sensitivity analysis in which one study at a time was removed and the rest were analyzed to evaluate whether the results could have been affected markedly by a single study.
